# Acknowledgement to Reviewers of *Antibiotics* in 2015

**DOI:** 10.3390/antibiotics5010009

**Published:** 2016-01-27

**Authors:** Antibiotics Editorial Office

**Affiliations:** MDPI AG, Klybeckstrasse 64, CH-4057 Basel, Switzerland; antibiotics@mdpi.com

The editors of *Antibiotics* would like to express their sincere gratitude to the following reviewers for assessing manuscripts in 2015. 

We greatly appreciate the contribution of expert reviewers, which is crucial to the journal’s editorial decision-making process. Several steps have been taken in 2015 to thank and acknowledge reviewers. Good, timely reviews are rewarded with a discount off their next MDPI publication. By creating an account on the submission system, reviewers can access details of their past reviews, see the comments of other reviewers, and download a letter of acknowledgement for their records. This is all done, of course, within the constraints of reviewer confidentiality. Feedback from reviewers shows that most see their task as a voluntary and mostly unseen work in service to the scientific community. We are grateful to our reviewers for the contribution they make.

Aires, AlfredoGupta, KajalPatel, TwishaAlmeida, C.Ha, Nam-ChulPaul O'Donnell, E.Aluwong, TagangHalton, KatePos, Klaas MartinusAndersson, Dan I.Harris, PatrickProctor, Richard A.Bania, JacekHirakawa, HidetadaRada, BalazsBartlett, Allison H.Hoffman, SebastienRao, KrishnaBarton, MaryHolmes, MarkRasschaert, GeertruiBavro, VassiliyHolzel, ChristinaReddy, Sujan C.Bjerrum, LarsHuang, AngelaRodríguez-Rojas, AlexandroBonomo, Robert A.Huigens III, Robert W.Roh, ChanghyunBrooks, Amanda E.Jantsch, JonathanRolek, KiriBuyle, F. M.Jeong, Seok HoonRoncada, PaolaCampos, JoséKahl, Barbara C.Sanchez-Amat, AntonioCarreno, Joseph J.Kao, DinaSauer-Budge, Alexis F.Castagner, BastienKenny, RachelSchaaper, Roeland M.Castellari, MassimoKimura, HideoSchulz, LucasChaptal, VincentKullar, RavinaSchwan, William R.Cherala, GaneshKwa, Andrea Lay-HoonSeiler, PeterChiu, Cheng-hsunKwon, SunghoonSimm, RogerChopra, TeenaLam, Jenny K.W.Sonawane, AvinashCiofu, OanaLarsson, Joakimstegmann, EviClaassen, EricLechardeur, DelphineStricker, Raphael BCole, AlexanderLeonard, AmaralTellez, GuillermoCoote, PeterLin, Hai-shuTenover, Dr. Fred C.Dancer, StephanieLuesch, HendrikTheriot, Casey M.Dandekar, ThomasMadaras-Kelly, KarlThibodeau, AlexandreDanziger, Larry HMartinez Lopez, AntonioVan Der Sijs, Heleende la Fuente-Nunez, CesarMaseda, Hideakivan Hoek, Angela H.A.M.Dhere, TanviMauel, Michael J.Vardakas, K. Z.Doernberg, SarahMccarter, Yvette S.Viale, PierluigiDomínguez Álvarez, EnriqueMcCoy, DorothyVindigni, Stephen MDrekonja, DimitriMcFarland, Lynne VVisschers, Vivianne H.M.Dubrovskaya, YaninaMena, AnaVitetta, LuisEschenauer, Gregory A.Mertz, DominikWagner, Gerd K.Fauvart, MaartenNagel, Jerod L.Wall, Geoffrey C.Feng, YangboNaughton, PatrickWeiss, DavidFernández, LucíaNayak, RajeshWilmes, MiriamFiol, NuriaNeethirajan, SureshWinkler, MalcolmFitzpatrick, MargaretNewman, Dianne KYan, AixinGoldberg, Joanna B.Nørskov-Lauritsen, N.Ying, ChingwenGomes, Maria SaloméOstrowsky, BelindaYu, DianaGovoni, Gregory R.Ouellette, AndreZhang, KaiGreenblatt, David J.Oughton, Matthew


